# Effect of Grape Seed Extract on Lipid Profile and Expression of Interleukin-6 in Polycystic Ovarian Syndrome Wistar Rat Model 

**DOI:** 10.22074/ijfs.2017.5007

**Published:** 2017-09-03

**Authors:** Zohreh Salmabadi, Homa Mohseni Kouchesfahani, Kazem Parivar, Latifeh Karimzadeh

**Affiliations:** 1 Department of Animal Biology, Faculty of Biological Sciences, Kharazmi University, Tehran, Iran; 2 Animal Center Laboratories and Cellular and Molecular Research Laboratory, Faculty of Biological Sciences, Kharazmi University, Tehran, Iran

**Keywords:** Dyslipidemia, Grape Seed Extract, IL-6, Polycystic Ovarian Syndrome, Wistar Rat

## Abstract

**Background:**

Polycystic ovary syndrome (PCOS) is a common but complex endocrine disorder and is the major cause of anovulation and consequent subfertility. In this study the effect of grape seed extract (GSE) on triglyceride (TG), total cholesterol (TC), highdensity lipoprotein-cholestrol (HDL-C), low-density lipoprotein-cholestrol (LDL-C) and interleukin-6 (IL-6) in PCOS Wistar rats were assessed.

**Materials and Methods:**

In this experimental study, 84 adult female Wistar rats were divided into 7 groups (n=12) including control (intact), Sham (estradiol valerate solvent injection), control PCOS and 4 experimental PCOS groups. To induce the syndrome, a single subcutaneous injection of 2 mg estradiol valerate was applied. In experimental groups, PCOS rats were treated with different doses of 50, 75, 100 and 200 mg/kg body weight (BW) GSE by intraperitoneal injection for 10 consecutive days. After harvesting blood serum, TG was measured by Glycerol-3-phosphate Oxidase-Peoxidase (GPO- PAP), TC by Cholesterol Oxidase-Peroxidase (CHOD-PAP), and HDL-C by sedimentation method, LDL-C by Friedwald calculation and IL-6 by ELISA method. The serum values of each parameter were analyzed using one-way ANOVA at P≤0.05.

**Results:**

In all experimental groups significant decrease of visceral fat was obvious as compared with control PCOS group. LDL-C, TC and IL-6 levels in experimental groups, particularly at dose of 50 mg/kg of GSE, were significantly decreased as compared with PCOS group. However, HDL-C levels were not significantly changed.

**Conclusion:**

: According to the findings of this study, it can be concluded that GSE with its effects on serum TC, LDL-C and IL-6 could reduce the effects of dyslipidemia and inflammation in PCOS rats and improve systemic symptoms of PCOS.

## Introduction

One of the most common endocrine disorders in women is polycystic ovarian syndrome (PCOS), affecting about 5 to 10% of women of reproductive age (15 to 45 years old) (1). PCOS was first described in 1935 by Stein and Leventhal (2). PCOS is a heterogeneous disease with a spectrum of endocrine protests such as polycystic ovarian morphology, ovarian follicular theca cell hyperplasia, chronic anovulation, menstrual disturbances and infertility. Common metabolic symptoms associated with this disease are obesity, hyperandrogenism, resistance to insulin and cardiovascular disorders. Women with PCOS demonstrate many features similar to metabolic syndrome, including dysfunction of the hypothalamic-pituitaryadrenal (HPA) axis, hyperinsulinemia, increase in cytokines and fat-derived factors and dyslipidemia (3).

Dyslipidemia in PCOS is characterized by increased triglycerides (TG) and decreased high density lipoprotein-cholesterol (HDL-C) (4). The classic criteria of atherogenic lipoprotein profile, characterized by elevated TG-rich lipoproteins, lower HDL levels, and higher low density lipoproteina (LDL)/HDL ratios, is the most distinctive characteristics of PCOS women, especially the obese ones (3). Besides conjunction of PCOS symptoms with those associated with metabolic syndrome, there is some evidence to present PCOS as a pro-inflammatory state (5). Blood levels of inflammatory markers, such as tumor necrosis factor-alpha (TNF-α), interleukin-6 (IL-6) and Creactive protein (CRP), are higher in women with PCOS than in controls matched for body mass index (BMI) and age (6). Most studies have reported a close relationship between levels of the inflammatory markers and insulin resistance/obesity, particularly central obesity (7). The location of IL-6 gene in humans is on the short arm of chromosome 7, and in mice it is on the proximal region of chromosome 5 (8). IL-6, a major proinflammatory cytokine, is produced in a variety of tissues, including activated leukocytes, adipocytes, and endothelial cells (9).

Grape is a plant growing throughout the world, and its ingredients and properties have been widely examined. One of the most abundant ingredients of grapes are phenolic compounds which are present in large amounts (10). Grape seed is one of the richest sources of polyphenols (11), which exhibit antioxidant, free radical scavenging properties, and lipid lowering effects (12). The most common polyphenols of grape seeds are procyanidins ranging in size from monomers to long-chain polymers, such as catechin, epicatechin, and procyanidin B2 (13). Because of its different properties, grape seed extract (GSE) has been proposed to be a good nominee for decreasing metabolic and cardiovascular changes related to obesity and metabolic disorders (14). United States Food and Drug Administration (FDA) in 2011 recognized grape seed and skin extracts to be safe, due to their health food ingredients (15). Relying on the fact that the antioxidant effects of GSE are 20 times greater than vitamin E and 50 times greater than vitamin C, the aim of this study was to determine the impacts of GSE on lipid profile and one of the main inflammatory markers, IL-6, in PCOS rat model.

## Materials and Methods

### Grape seed extract preparation

Red grape (Vitis vinifera) was obtained from the city of Arak (Iran) and then washed and dried. Seeds were separated from grapes and were ground in a grinder (Shimaz, Iran). The powdered grape seeds (75 g) were added to 200 ml of 70% ethanol and were maintained in incubator (Fanazmagostar, Iran) for 24 hours at 40°C, rotated daily for three hours on a rotator device at 200 rpm and filtered by a filter paper Whatman No. 1. The solvent was then removed using a rotary evaporator (Hoilph, German). This procedure was repeated three times, and all collected samples were kept at -20°C. Shortly before each experiment 50, 75, 100 and 200 mg/kg of the dry extract was dissolved in 0.9% normal saline as solvent (Cytomatin gene, Iran).

### Animals

In this experimental study, 84 female Wistar rats weighing 160 ± 20 g were used. Animals were kept in the animal maintenance and breeding center of Kharazmi University, in special cages under appropriate environmental conditions and desired temperature of 20-24°C, in 12-hours light/dark cycles and with free access to food and water. Rats with a 2-3 regular estrous cycles during the twelve to fourteen days of vaginal smear, and in the estrous phase of their reproductive cycle were chosen for experiments. To induce PCOS phenotype a variety of hormonal and non-hormonal techniques exist including treatments with testosterone, estradiol valerate (EV), dehydroepiandrosterone (DHEA), adrenocorticotropic hormone (ACTH) or long-term use of light. In this study hormonal induction of PCOS by EV (Aburaihan Co., Iran) was used. Rats were divided into 7 groups (n=12) including control (intact), sham (estradiol valerate solvent injection), control PCOS and 4 experimental PCOS groups. PCOS was induced by a single subcutaneous injection of 2 mg EV. Sham group received a similar dose of sesame oil as a solvent of EV and the control group had no injections. Successful induction of the syndrome was achieved by eight weeks showing symptoms such as irregular estrous cycle and occurrence of the persistent vaginal cornification (PVC) phase. After ensuring that the syndrome was induced, PCOS rats were divided into 5 groups, named as control PCOS group, and 4 experimentals (n=12 each). The experimental groups received 50, 75, 100 and 200 mg/kg body weight (BW) GSE by intraperitoneal injections for 10 consecutive days. Five days after the last injection, rats of all groups were sacrificed with carbon dioxide inhalation and their blood was taken off from left ventricle and serum samples were separated by centrifugation at 6,000 rpm for five minutes. Samples were stored at -20°C prior to examining the expression of IL-6 and lipid profile.

### Lipid profile measurements

After 10 hours of fasting, 5 ml of rat blood was collected in sterile bottles and allowed to clot for about an hour at 37°C. Then serum was separated and stored at -20°C. The serum level of TG were evaluated by the Glycerol-3-phosphate Oxidase-Peoxidase (GPO-PAP), as End Point Assay. Total cholesterol (TC) by Cholesterol Oxidase-Peroxidase (CHODPAP) and the HDL-C level was determined after lipoproteins were precipitated .The LDL-C level was by the Friedewald’s equation (16).

VLDL=TG/5

LDL=TC-HDL-VLDL

### IL-6 assay

The amount of IL-6 in blood serum was measured by ELISA using a rat IL-6 platinum ELISA kit (Bender Medsystems, Austria). The sensitivity of the assay for IL-6 was 12 pg/mL.

### Statistical analysis

All data were presented as mean ± SE. Statistical significance was evaluated with one-way analysis of variance (ANOVA) using SPSS18. Significant differences between groups were measured using Tukey tests. P≤0.05 was considered significant and relevant histograms were drawn by the EXCEL program.

### Ethical considerations

All research animals were treated in compliance with the guidelines for the care and use of animals approved by our institutions in accordance with the principles of laboratory animal care (NIH Guide for the Care and Use of Laboratory Animals, Institute of Laboratory Animal Resources, National Research Council, Washington, D.C.) (code: No. 616.18)

## Results

**Fig.1:**
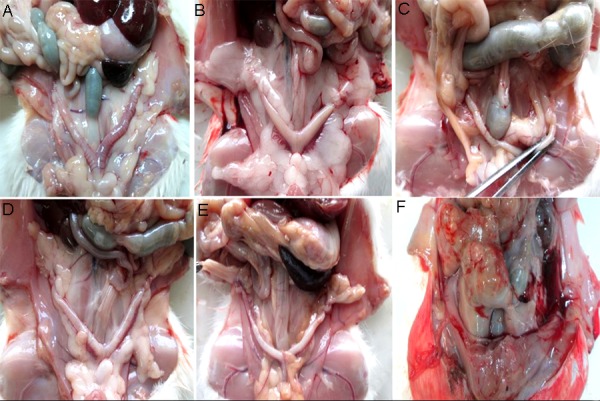
Ovary morphology showing decrease in visceral fat in the grape seed extract (GSE) treated groups compared with control and polycystic ovary syndrome (PCOS). Fat tissue in the abdominal cavity, particularly around the uterus and ovaries decreased in PCOS treatment groups. **A.** Control group, **B.** PCOS group, **C.** PCOS group treated with 50 mg/kg GSE, **D.** PCOS group treated with 75 mg/kg GSE, **E.** PCOS group treated with 100 mg/kg GSE, and **F.** PCOS group treated with 200 mg/ kg GSE.

In all 4 experimental PCOS groups treated with GSE, notable reduction of visceral fat was observed as compared to sham, control and control PCOS groups (Fig .1). In the PCOS group treated with 200 mg/kg GSE, a severe inflammation of abdominal cavity and intensive changes in the appearance of liver and abdominal cavity was observed, indicating the destructive effects of the high dose applied.

**Fig.2:**
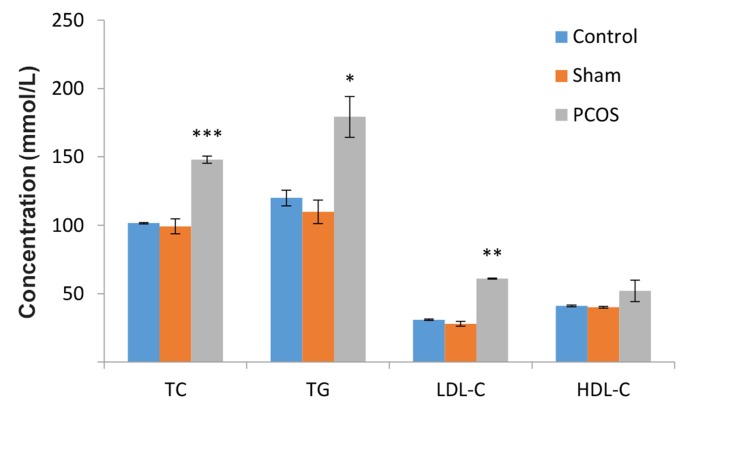
Comparison of lipid profile levels in polycystic ovary syndrome (PCOS) group as compared to the sham and control groups. The serum level of TC, TG and LDL-C in PCOS compared with sham and control groups have shown significant increases. TC; Total cholestrol, TG; Triglyceride, LDL-C; Low-density lipoprotein- cholestrol, HDL-C; High density lipoprotein-cholestrol, *; P<0.05, **; P<0.01, and ***; P<0.001 compared with control and sham (treated with 0.9% normal saline).

As shown in Figure 2, a significant increase in the serum levels of TC, TG and LDL-C, but not HDLC, was observed in PCOS group as compared to the sham and control groups (P≤0.05).

**Fig.3:**
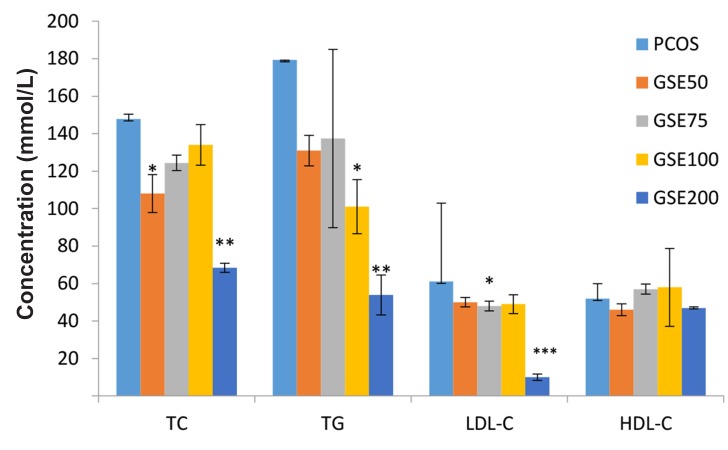
Comparison of the lipid profile levels in grape seed extract (GSE) treated groups with polycystic ovary syndrome (PCOS) group. Lipid profile showed decrease in GSE groups compared with PCOS. TC; Total cholestrol, TG; Triglyceride, LDL-C; Low density lipoprotein- cholestrol, HDL-C; High density lipoprotein-cholestrol, GSE50; PCOS treated with a dose of 50 mg/kg GSE, GSE75; PCOS treated with a dose of 75 mg/kg GSE, GSE100; PCOS treated with a dose of 100 mg/kg GSE, GSE200; PCOS treated with a dose of 200 mg/kg GSE, *; P<0.05, **; P<0.01, and ***; P<0.001 compared with PCOS.

Concentration of TC was significantly lowered in the GSE50 and GSE200 groups as compared with control PCOS group. On the other hand, TG plasma concentration was significantly decreased in the GSE100 and GSE200 groups compared to the control PCOS group. LDL-C level was significantly reduced in the 75 and 200 mg/kg GSE as compared with control PCOS group (P≤0 .001). Comparison of HDL-C levels did not show significant differences between GSE treated groups and PCOS group (Fig .3).

**Fig.4:**
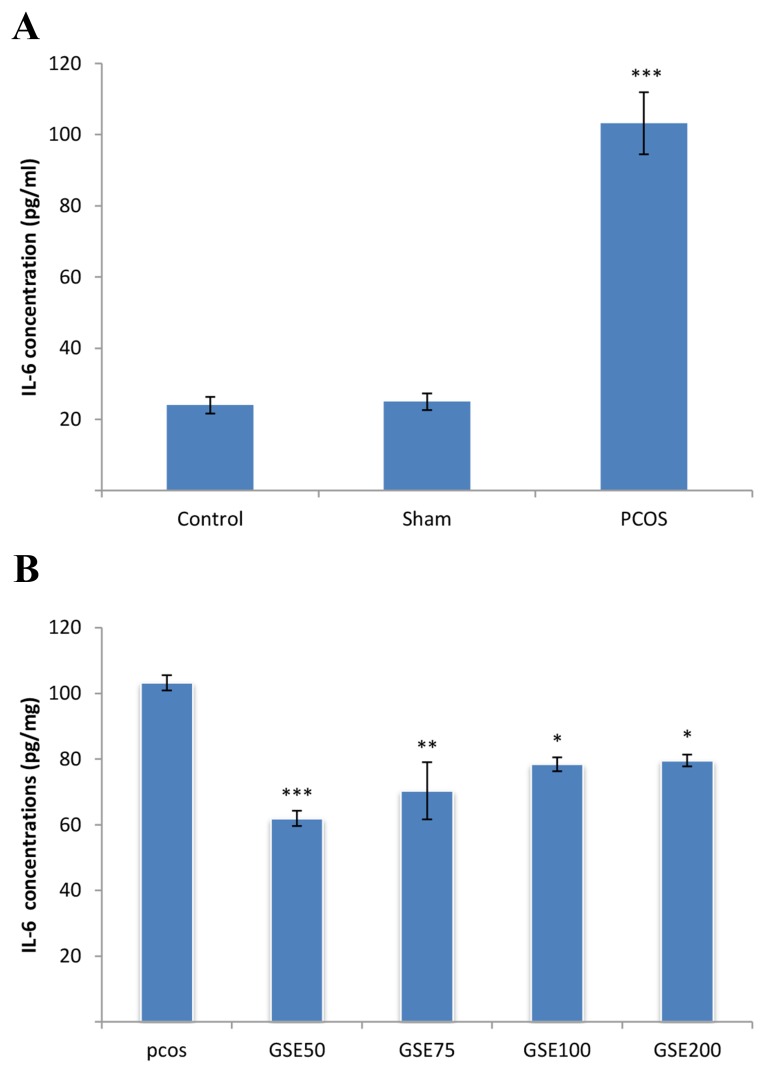
Comparison of interleukin-6 (IL-6) levels between groups. A. Serum interleukin-6 (IL-6) concentrations showed significant increase in polycystic ovary syndrome (PCOS) group compared to the control and sham groups and B. Comparison of IL-6 levels in grape seed extract (GSE) treated groups with PCOS group. A significant reduction in GSE treated groups were observed as compared with PCOS group. GSE50; PCOS treated with a dose of 50 mg/kg GSE, GSE75; PCOS treated with a dose of 75 mg/kg GSE, GSE100; PCOS treated with a dose of 100 mg/kg GSE, GSE200; PCOS treated with a dose of 200 mg/kg GSE, *; P<0.05, **; P<0.01, and ***; P<0.001.

In Figure 4A a significant increase in the amount of IL-6 in control PCOS group compared to the control and Sham groups was observed (P<0.001). By comparing PCOS groups treated with different doses of GSE with control PCOS group, a significant decrease in IL-6 level was observed (Fig.4B).

## Discussion

PCOS is related to various patterns of dyslipidemia including reduced HDL-C, high levels of TG, TC and LDL-C (17). Our analysis of serum lipids showed increase in the TC, TG and LDL-C levels after the induction of PCOS by EV. Abdominal fat accumulation has been observed in about half of PCOS patients (18). Obesity is a classic characteristic of PCOS, with 30-60% of patients being overweight to some degree (19). Increased abdominal fat has been linked to insulin resistance and increased cardiovascular risk. Because many patients with PCOS present abdominal obesity, it may be the cause of insulin resistance seen in PCOS (20). In the present study, similar to previous studies, an increase in external visceral fat in PCOS rats was observed. IL-6 modulates the action of aromatase, a key regulatory enzyme for estrogen metabolism (21); The release of IL-6 into the systemic circulation and the fact that this release is greater in obese subjects support a possible novel role for IL-6 as a systemic regulator of BW (an adipostat) and a regulator of lipid metabolism. IL-6 receptors are present in the hypothalamus, which also supports the idea that this cytokine has direct central actions (22).

Dyslipidemia, type 2 diabetes and cardiovascular disorders and the link between these conditions has been assumed to be chronic inflammation. Visceral obesity has been defined as a state of low-grade inflammation because visceral adipose tissue is able to produce cytokines (TNF-α, IL-6, and IL-1), chemokines (IP-10, IL-8, IL-18, monocyte chemotactic protein-1 (MCP-1), and regulated on activation normal T expressed and secreted (RANTES), and other adipokines, free fatty acid (FFA), plasminogen activator-1 (PAI-1, leptin, resistin, visfatin, and adiponectin) that act, directly or indirectly, as mediators of systemic inflammation (23). Linscheid et al. (24) showed that adipose tissue emerged as an important source of pro-inflammatory mediators including TNF-α, IL- 6, and procalcitonin (ProCT). Results of IL-6 in the present study are in accordance with that of Kershaw and Linscheid.

Studies of Charradi et al. (14) showed that GSE is a safe anti-obesity and cardioprotective agent that should also have potential benefits in other inflammatory damaging conditions like stroke. Epidemiological studies report an inverse association between GSE consumption and mortality from cardiovascular diseases (25). In numerous studies, flavonoids and their derivatives have been reported to reduce LDL oxidation in both humans and animal models (26). Studies using flavonoids have also shown reductions in plasma lipids and multiple effects on lipoprotein metabolism (27). GSE prevents the differentiation of adipocytes *in vitro* (28). In the present study, all doses of 50, 75, 100, 200 mg/kg GSE decreased visceral fat in the treated rats. However, with lower doses of (50, 75 mg/kg) GSE, appearance of the ovary tissue was normal. Measuring the granulosa layer thickness in PCOS groups treated with GSE revealed significant increase as compared with the control PCOS group. The diameter of theca layer of antral follicles in PCOS groups treated with GSE at doses of 50 and 75 mg/kg showed significant decrease as compared with the control PCOS group.

We have previously shown that in doses of 50, 75, 100 mg/kg of GSE, the number of small follicles, antral and Graafian follicles, and in all 4 doses the number of corpus luteum has significantly increased, indicating a dramatic improvement in the polycystic ovaries (29). Since GSE at a dose of 200 mg/kg caused remarkable visceral inflammation, accumulation of fluid in the peritoneal cavity and severe damages to various organs (especially the liver), at this dose it was considered as toxic, and two doses of 50 and 75 mg/kg GSE due to their improving effects on systemic PCOS symptoms were considered as effective doses. Grape seeds possess cardioprotective effects by alleviating inflammatory conditions and reducing oxidative stress (30). Besides the free radical scavenging and antioxidant activity, pro-anthocyanidins exhibit vasodilatory, anti-carcinogenic, anti-allergic, anti-inflammatory, anti-bacterial, cardioprotective, immune stimulating, anti-viral and estrogenic activities, as well as being inhibitors of the enzymes phospholipase A2, cyclooxygenase and lipooxygenase (31).

Schewe et al. (32) showed that GSE has antiinflammatory properties. Terra et al. (33) showed that orally ingested GSE helps preventing imbalanced cytokine patterns. Polyphenols in GSE could therefore be responsible for an anti-inflammatory effect in experimental studies (34). Terra et al. (33) demonstrated that induction of IL-6, CRP, and TNF-α expressions by high fat diet were reduced by adding procyanidins extract to the diet. They also showed that procyanidins reduced macrophage level. So, the inhibition of the cytokine expression in adipose tissue might be due to a decrease in the number of macrophages, but procyanidins mayalso directly affect the proinflammatory pathways in both adipocytes and macrophages. In any case, these findings demonstrate the potential effects of procyanidins on such low-grade inflammation-related diseases as obesity.

In an *in vitro* study by Moreno, GSE showed the inhibitory effects on fat metabolizing enzymespancreatic lipase and lipoprotein lipase activities and on lipolysis of 3T3-L1 murine adipocytes. This inhibiting activity suggests that GSE might be useful as a treatment to limit dietary fat absorption and the accumulation of fat in adipose tissue (35). Grape seeds contain numerous polyphenols including resveratrol and quercetin and have been used in an effort to treat conditions that comprise metabolic syndrome. Pigs fed by Resveratrol at a dose of 100 mg/kg per day for 7 weeks had lower serum glucose, cholesterol, LDL, systolic blood pressure, and BMI (36). In the present study, after induction of PCOS, an increase in the visceral fat and expression of IL-6 in animals was occurred, while by using GSE, the level of IL-6, a marker of inflammation, has been significantly reduced.

## Conclusion

According to the results of this study, it can be concluded that treatment with GSE causes significant decrease in visceral fat, cholesterol, TG, LDL-C and IL-6. Since the adipose tissue produces the IL-6, treating with GSE might be helpful for reducing adipose tissue, which is the main source of IL-6. By lowering the levels of IL-6, cholesterol, TG, LDL-C, dyslipidemia and inflammatory symptoms of PCOS will be improved.
